# Role of Autophagy in the Control of Cell Death and Inflammation

**DOI:** 10.4110/in.2009.9.1.8

**Published:** 2009-02-28

**Authors:** Myung-Shik Lee

**Affiliations:** Department of Medicine, Samsung Medical Center, Sungkyunkwan University, School of Medicine, Seoul 135-710, Korea.

**Keywords:** autophagy, inflammation, immunity, apoptosis, necrosis

## Abstract

There is mounting evidence that autophagy is involved in diverse physiological and pathological processes that have immense relevance in human development, diseases and aging. Immunity and inflammation are not exceptions. Here, the role of autophagy in the control of immune processes particularly that related to cell death and inflammation is discussed.

## INTRODUCTION

Macroautophagy (here referred to as autophagy) is a process of sequestration and removal of damaged organelles/proteins for recycling of their constituents and supply of nutrients. In addition, autophagy provides signals for the removal of apoptotic cells and genomic stability (1). Hence, autophagy can be generally considered as a protective measure for cells against various types of injuries or from continuous cellular wear-and-tear. The physiological role of autophagy in the mammalian system was demonstrated in several *in vivo* animal models. Disruption of autophagic processes leads to failure of cavitation during embryogenesis (2) or accumulation of abnormal mitochondria in adult tissues (3). In addition to the physiological roles, dysregulated autophagy has been suggested to play pathogenic roles in a variety of disease processes including neurodegeneration (4,5) and cardiomyopathy (6) particularly when cellular stress is increased, which is likely to be due to accumulation of damaged molecules and organelles (6). Other studies revealed important roles for autophagy in tumor suppression (1) and ER stress responses (7).

## AUTOPHAGY AND CELL DEATH

As discussed above, autophagy plays a protective role in diverse types of cellular stress. Paradoxically, autophagy can also lead to a form of non-apoptotic cell death known as "type 2 programmed cell death" (8-10) particularly when autophagy is excessive (11). Thus, the dual nature of autophagy can either promote cell death or protect cells from diverse types of injuries depending on the cellular and environmental context. In mice models that have a deficiency of autophagy specifically in neurons, increased apoptosis of neurons were observed (4,5). We and others have shown in mice with a pancreatic β-cell-specific deletion of an essential autophagy gene (*Atg7*) that the pancreatic β-cell mass was decreased due to increased pancreatic β-cell death and decreased β-cell proliferation leading to a reduction of insulin production and hyperglycemia (12,13). Large intracellular aggregates containing ubiquitin and p62 were observed in the pancreatic islets of β-cell-specific *Atg7*-null mice (13,14). However, inflammation was not observed but in the case of hepatocyte-specific *Atg7*-null mice it was observed (data not shown). Increased pancreatic β-cell death in β-cell-specific *Atg7*-null mice is consistent with previous data that suggests autophagy plays a protective role against cell injury and autophagy-deficient cells would be susceptible to diverse types of cell death. Several potential mechanisms of increased susceptibility to cell death were proposed such as a shortage of bioenergetic sources like NADPH_2_ and ATP. The shortage of these sources may trigger apoptosis presumably through a direct effect on the mitochondria or de-inhibit the activation of caspases or permeabilization of the mitochondrial outer membrane (15) or through activation of p27 by metabolic stress and energy deficiency that may also lead to cell cycle arrest and decreased proliferation (16). Modulation of mitochondrial mass or cytochrome *c* content by autophagy may be related to the changes in the susceptibility to apoptotic inducers (17). However, detailed mechanism of increased pancreatic β-cell death in β-cell-specific *Atg7*-null mice is unknown. Furthermore, it was suggested that the real process inhibited by autophagy is necrosis rather than apoptosis which may be of relevance to the control of cell death-associated inflammation and tumorigenesis (18).

## AUTOPHAGY, MACROPHAGE AND INFLAMMATION

Autophagy may also play a crucial role in the regulation of inflammation and immunity. For instance, autophagy has been reported to be critically involved in the eradication of microbes such as *Mycobacterium tuberculosis* (19), antigen presentation by professional antigen-presenting cells (APC) (20) and tolerance induction in T cells (21). Autophagy may also regulate inflammation or immunity by modulating survival or death of lymphocytes (22) and APC such as macrophages. We have reported the role of ROS and STAT1 in the necrotic death of macrophage by LPS in the presence of zVAD, a pan-caspase inhibitor (23). Then, a role for autophagy in this type of macrophage death was reported. Xu et al. showed the death of macrophages by LPS in combination with zVAD had microscopic features of autophagy such as punctuate LC3 pattern and formation of autophagosomes surrounded by double membranes (24). Furthermore, Yu et al. reported that autophagy selectively degraded catalase leading to the accumulation of ROS and non-apoptotic death of macrophages (25). Contrary to those reports, a protective role of autophagy in macrophage death by zVAD was also reported (26). If zVAD is able to inhibit cathepsin and lysosomal function as reported, then the apparent increased conversion of LC3-I to LC3-II and the accumulation of autophagosomes may be due to the blockade of autophagy at the lysosomal step rather than to an increased autophagic activity. Such a theory may provide a clue to the long-sought question regarding the additional target molecules of zVAD in addition to caspases. Thus, it is still unclear whether autophagy protects macrophages from death or induces macrophage death in the presence of zVAD.

Autophagy may also modulate functions of macrophages in addition to their survival or death. LC3 recruitment was observed when macrophages ingested latex beads associated with TLR ligands, but not without TLR ligands (27). However, classical autophagosomes with double membranes were not observed in that case. Furthermore, microbes engulfed by autophagy-deficient macrophages showed a marked survival advantage suggesting that TLR signaling may usurp the autophagic machinery to deliver phagocytic material to the lysosomal pathway (27). TLR agonists themselves have been reported to activate the autophagic pathway (28); however, a recent paper reported contradictory results (29).

## AUTOPHAGY AND INFLAMMATORY/IMMUNE DISEASES

Besides *in vitro* or animal models, direct involvement of the autophagy-inflammation relationship in human diseases was recently reported. Crohn's disease is characterized by chronic mucosal or transmural inflammation in the small intestines particularly in the distal ileum. Several theories have been proposed related to the pathogenesis of Crohn's disease such as mutations of NOD_2_, an important intracellular innate immune receptor (30). Also several animal models are available for the study of Crohn's disease. However, the detailed molecular pathogenic mechanism is still far from clear. Large-scale genetic analysis revealed more than 30 loci showing linkage to the disease development process (31). Among them, Atg16L1 locus is of particular interest because Atg16L1 is a gene homolog to yeast Atg16, an essential gene for autophagy in yeast. Atg16L1 is an integral component of the large molecular weight complex comprising Atg12-Atg5 conjugate and has been suggested to play an important role in the conjugation of LC3 (Atg8) to phosphatidylethanolamine. Thus, Atg16L1 has been regarded as a key gene that may provide a clue to the relationship between autophagy and inflammation in human diseases. Indeed, a recent paper showed a role of Atg16L1 in the control of inflammatory cytokines from macrophages (29). Release of cytokines such as IL-1β and IL-18 after LPS or infection with non-invasive bacteria such as *E. coli* was significantly increased in *Atg16L1*-null macrophages. Intriguingly, IL-1β release after infection with invasive bacteria such as *Salmonella typhimurium* was not increased in *Atg16L1*-null macrophages. Increased IL-1β or IL-18 secretion from *Atg16L1*-null macrophages appears to be due to increased ROS production inducing activation of caspase-1. However, the molecular details leading to the increase ROS production in autophagy-deficient macrophages are still unknown. Mice lacking Atg16L1 in hematopoietic cells did not show spontaneous colitis; however, they showed much severer colitis after dextran sulphate sodium (DSS) challenge. Another recent paper showed an abnormality in Paneth cells that are specialized cells in the intestinal epithelium secreting antimicrobial peptides or other intestinal peptides (32) in Atg16L1-deficient mice (33). Furthermore, production of adiponectin and leptin that modify the intestinal environment was also altered in autophagy-deficient Paneth cells.

## CONCLUSION

Autophagy appears to be critically involved in inflammation and immunity. So far, several pioneering studies have been done. Some of the results are not consistent with each other, which are more than likely due to an incomplete knowledge or misconception regarding the process of autophagy. For example, it is becoming clear that autophagy level and autophagic activity are not always the same, and the blockade of the autophagic process may be confused with increased autophagy due to the possible accumulation of autophagosomes. Thus, more thorough evaluation will be required for proper interpretation of future experimental findings and also for that of previously reported results. More investigations are being done and will be necessary to reveal the relationship between autophagy and the diverse types of inflammatory and (auto) immune disorders which will lead to the elucidation of hitherto unknown and unsuspected aspects of both adaptive and innate immunity.
